# Reliability Analysis of the New Exponential Inverted Topp–Leone Distribution with Applications

**DOI:** 10.3390/e23121662

**Published:** 2021-12-10

**Authors:** Ahmed Sayed M. Metwally, Amal S. Hassan, Ehab M. Almetwally, B M Golam Kibria, Hisham M. Almongy

**Affiliations:** 1 Department of Mathematics, College of Science, King Saud University, Riyadh 11451, Saudi Arabia; dalsayed@ksu.edu.sa; 2 Department of Mathematical Statistics, Faculty of Graduate Studies for Statistical Research, Cairo University, Giza 12613, Egypt; amal52_soliman@cu.edu.eg; 3 Department of Statistics, Faculty of Business Administration, Delta University of Science and Technology, Gamasa 11152, Egypt; 4 Department of Mathematics and Statistics, Florida International University (FIU), 11200 SW 8th St, Miami, FL 33199, USA; kibriag@fiu.edu; 5 Department of Applied Statistics and Insurance, Faculty of Commerce, Mansoura University, El-Mansoura 35516, Egypt; elmongyh@mans.edu.eg

**Keywords:** new exponential-X, stress–strength reliability, entropy, Bayesian, maximum product spacing

## Abstract

The inverted Topp–Leone distribution is a new, appealing model for reliability analysis. In this paper, a new distribution, named new exponential inverted Topp–Leone (NEITL) is presented, which adds an extra shape parameter to the inverted Topp–Leone distribution. The graphical representations of its density, survival, and hazard rate functions are provided. The following properties are explored: quantile function, mixture representation, entropies, moments, and stress–strength reliability. We plotted the skewness and kurtosis measures of the proposed model based on the quantiles. Three different estimation procedures are suggested to estimate the distribution parameters, reliability, and hazard rate functions, along with their confidence intervals. Additionally, stress–strength reliability estimators for the NEITL model were obtained. To illustrate the findings of the paper, two real datasets on engineering and medical fields have been analyzed.

## 1. Introduction

There are several univariate continuous distributions in the present statistical literature that may be used in a range of data modeling applications. However, it appears that the many distributions that are available are insufficient to manage the diverse data encountered in fields such as medicine, engineering, demography, biology, actuarial science, economics, finance, and reliability. Statistical and applied researchers are interested in constructing new extended continuous distributions that are more effective for data modeling. Adding parameters, compounding, generating, transformation, and composition are all methods for extending well-known distributions.

In the last couple of decades, the generation of new families of continuous distributions has attracted several statisticians to develop new models. Our interest is particularly in a new family proposed by Huo et al. [1] called the new exponential-X (NE-X) family. The cumulative distribution function (CDF) and probability density function (PDF) of the NE-X family are defined as:

(1) $$F\left(x;\theta ,\zeta \right)=1-{\left[\frac{1-G{\left(x;\zeta \right)}^{2}}{1-\left(1-\theta \right)G{\left(x;\zeta \right)}^{2}}\right]}^{\theta },$$

and

(2) $$f\left(x;\theta ,\zeta \right)=2\theta^{2}g\left(x;\zeta \right)G\left(x;\zeta \right)\frac{{\left[1-G{\left(x;\zeta \right)}^{2}\right]}^{\theta -1}}{{\left[1-\left(1-\theta \right)G{\left(x;\zeta \right)}^{2}\right]}^{\theta +1}},$$

where g(x;ζ) and G(x;ζ) are the PDF and CDF, respectively, for any baseline distribution with the set of parameters ζ. The set of parameters ζ can contain more than one parameter according to the type of distribution, and θ is a parameter of NE-X family where θ>0.

Inverted or inverse distributions are important in many fields, including biological sciences, life test problems, chemistry data, medical sciences, and so on, because of their applicability. Inverted conformation distributions have a different structure than non-inverted conformation distributions in terms of density and hazard functions. The reader can consult Barco et al. [2], Abd AL-Fattah et al. [3], Hassan and Abd-Allah [4], Hassan and Mohamed [5], Muhammed [6], Hassan and Mohamed [7], Almetwally [8], and Hassan and Nassr [9] for discussions and applications of inverted distributions.

The inverted Topp–Leone (ITL) distribution with only one shape parameter (δ≥0), which was presented by Hassan et al. [10], is a recent, significant model among the well-known inverted distributions. It density and hazard functions take different shapes according to value of δ, including unimodal, right skewed, increasing, decreasing, and upside down. The PDF and CDF of the ITL distribution are specified, respectively, as follows:

(3) $$G\left(x;\delta \right)=1-\frac{{\left(1+2x\right)}^{\delta }}{{\left(1+x\right)}^{2\delta }};\;\;\;\;\;\;\;x\geq 0,\;\delta >0,$$

and,

(4) $$g\left(x;\delta \right)=2\delta x\frac{{\left(1+2x\right)}^{\delta -1}}{{\left(1+x\right)}^{2\delta +1}};\;\;\;\;\;x,\delta >0.$$

Some authors studied and developed new extensions and generalizations of the ITL distribution, such as the power ITL distribution prepared by Abushal et al. [11], Kumaraswamy ITL distribution introduced by Hassen et al. [12], alpha power ITL distribution presented by Ibrahim et al. [13], modified Kies ITL distribution introduced by Almetwally et al. [14], odd Weibull ITL distribution suggested by Almetwally [15], and half logistic ITL distribution prepared by Bantan et al. [16].

In this paper, a new ITL distribution based on the NE-X family is proposed. We call it the new exponential ITL (NEITL) distribution. Our motivations for presenting the NEITL distribution are as follows: (i) to increase the flexibility of the ITL distribution for modeling several types of data; (ii) to allow researches to obtain more flexible density and hazard rate functions; (iii) real-world examples from medical, engineering, and other fields demonstrate that the NEITL model outperforms other competing distributions, justifying its implementation in these domains.

Another motivation for the present study was estimating the NEITL parameters, reliability function, and hazard rate function using three estimation methods to recommend the best estimates via a simulation study. The suggested procedures are maximum likelihood (ML), maximum product of spacing (MPS), and Bayesian procedures. The asymptotic and bootstrap confidence intervals are shown. Furthermore, we obtained the stress–strength (S–S) reliability estimator assuming that both the strength $\left(X_{1}\right)$ and stress $\left(X_{2}\right)$ have NEITLs with different shape parameters. In simulation research, statistical analysis was performed between these methods to assess their effectiveness and to investigate how these estimators function for various sample sizes and parameter values. Two applications showed that the NEITL distribution provides a better fit than some other distributions.

The rest of this essay is presented as follows. In Section 2, we define the NEITL distribution. Some of the statistical features of the NEITL distribution are determined in Section 3. The NEITL distribution’s reliability, hazard function (HF), and S–S reliability are covered in Section 4. Section 5 considers point estimate, asymptotic, and bootstrap confidence intervals utilizing ML, Bayesian, and MPS estimation methods. A simulation experiment is presented in Section 6 to compare the performances of the estimates presented in Section 5. Data implementations are explored in Section 7. The article is closed with some conclusions.

## 2. NEITL Distribution

**Figure 1:**
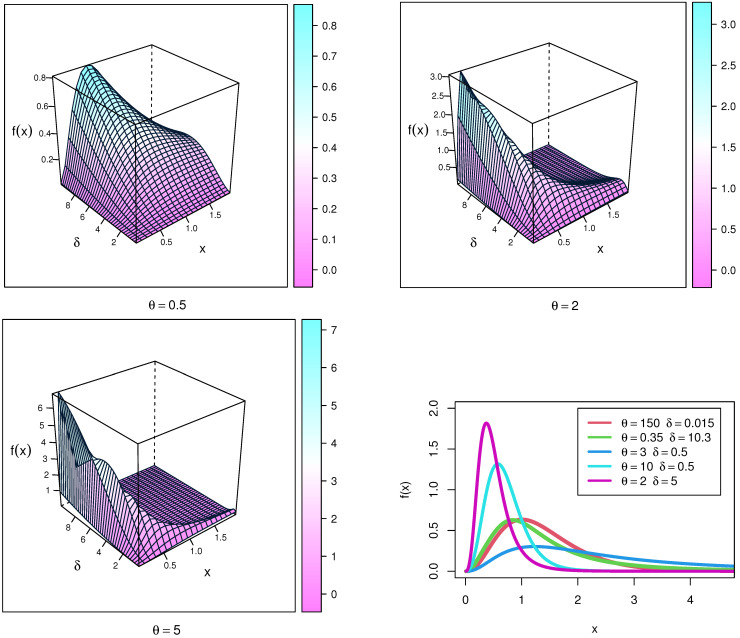
PDFs plots of the NEITL distribution.

The two-parameter NEITL distribution is a special model of the NE-X family with the ITL distribution as a baseline function. We get the CDF and PDF of the NEITL distribution by replacing the CDF and PDF of the ITL model in (1) and (2), respectively.

(5) $$F\left(x;\Omega \right)=1-{\left[\frac{1-{\left[1-\frac{{\left(1+2x\right)}^{\delta }}{{\left(1+x\right)}^{2\delta }}\right]}^{2}}{1-\left(1-\theta \right){\left[1-\frac{{\left(1+2x\right)}^{\delta }}{{\left(1+x\right)}^{2\delta }}\right]}^{2}}\right]}^{\theta };\;x>0,\;\theta ,\delta >0,$$

and

(6) $$\;f\left(x;\Omega \right)=4\theta^{2}\delta x\frac{{\left(1+2x\right)}^{\delta -1}}{{\left(1+x\right)}^{2\delta +1}}\left[1-\frac{{\left(1+2x\right)}^{\delta }}{{\left(1+x\right)}^{2\delta }}\right]\frac{{\left(1-{\left[1-\frac{{\left(1+2x\right)}^{\delta }}{{\left(1+x\right)}^{2\delta }}\right]}^{2}\right)}^{\theta -1}}{{\left(1-\left(1-\theta \right){\left[1-\frac{{\left(1+2x\right)}^{\delta }}{{\left(1+x\right)}^{2\delta }}\right]}^{2}\right)}^{\theta +1}};\;x>0,\;\theta ,\delta >0,$$

where Ω is a vector of parameters (δ,θ) for this distribution. Figure 1 visually displays the PDF plots and 3-D plots of X using NEITL and parameters (Ω). The NEITL distribution may be right-skewed and unimodal, according to the PDF plots.

Furthermore, we obtain an explicit linear representation of the density and distribution functions by using the generalized binomial expansion. Hence, for k>0, the *k* is a real non-integer, and for z<1 we use the following expansion with negative power:

(1−z)−k=∑i=0∞i+k−1k−1zi.

Additionally, we use the binomial expansion below with positive power:

(1−w)v=∑j=0∞vj(−1)jwj.

Let $z=\left(1-\theta \right)G{\left(x;\zeta \right)}^{2}$, and $w=G{\left(x;\zeta \right)}^{2}$. Then, the linear representation of CDF for NE-X family (1) is given by

(7) F(x;Ω)=1−∑i,j=0∞i+θ−1θ−1θj(−1)j(1−θ)iG(x;ζ)2(i+j).

By applying the previous expansion (7) on ITL distribution (3), we have CDF of the NEITL distribution in an expanded form as the following:

(8) F(x;Ω)=1−∑i,j=0∞i+θ−1θ−1θj(−1)j(1−θ)i1−(1+2x)δ(1+x)2δ2(i+j),

and let u=2(i+j), and v=θ−1. Then, the CDF of the NEITL distribution can be rewritten as follows:

(9) F(x;Ω)=1−∑i,j=0∞∑l=0ui+vvθjul(−1)j+l(1−θ)i(1+2x)lδ(1+x)2lδ.

Hence, the CDF (9) is represented as an infinite linear combination of the ITL distribution function with parameter lδ.

In addition to the expression (9), we derive PDF expression of the NEITL distribution distribution as follows:

Let $z=\left(1-\theta \right)G{\left(x;\delta \right)}^{2}$. Then the linear representation of PDF for NE-X family (2) is given by

(10) f(x;Ω)=2θ2∑i,j=0∞i+θθvj(−1)j(1−θ)ig(x;δ)G(x;δ)u+1.

Using CDF (3) and PDF (4) in (10), and binomial expansion, we obtain the following expansion:

(11) $$f\left(x;\Omega \right)=2\delta x\sum_{i,j=0}^{\infty }\sum_{l=0}^{u+1}K_{i,j,l}\frac{{\left(1+2x\right)}^{\delta (l+1)-1}}{{\left(1+x\right)}^{2\delta (l+1)+1}},$$

where Ki,j,l=2θ2l+1i+θθvju+1l(−1)j+l(1−θ)i. It is the PDF function of the ITL distribution with parameter δ(l+1).

## 3. Mathematical Properties

Here, some structure properties of the NEITL distribution are investigated, such as ordinary and incomplete moments, the quantile function and random number generation, Rényi and ρ-entropies, and the S–S reliability model.

### 3.1. Ordinal Moments

The *r*th moment of the NEITL distribution is given by

(12) $$\begin{array}{cc} E(X^{r}) & =2\delta \sum_{i,j=0}^{\infty }\sum_{l=0}^{u+1}K_{i,j,l}\int_{0}^{\infty }x^{r+1}\frac{{\left(1+2x\right)}^{\delta (l+1)-1}}{{\left(1+x\right)}^{2\delta (l+1)+1}}dx\\ & =2\delta \sum_{i,j=0}^{\infty }\sum_{l=0}^{u+1}K_{i,j,l}\int_{0}^{\infty }x^{r+1}\frac{{\left(1+\frac{x}{x+1}\right)}^{\delta (l+1)-1}}{{\left(1+x\right)}^{\delta (l+1)+2}}dx. \end{array}$$

Using the binomial expansion in (12), we can define (1+z)δ(l+1)−1=∑q=0∞(δ(l+1)−1q)zq. Let $z=\frac{x}{x+1};\;\;0<z<1$. Then the *r*th moment of the NEITL distribution has the form

(13) $$\begin{array}{cc} E(X^{r}) & =2\delta \sum_{i,j,q=0}^{\infty }\sum_{l=0}^{u+1}\tau_{i,j,l,q}\;\;\beta \left(r+q+2,\delta (l+1)-r\right), \end{array}$$

where $\beta \left(a,b\right)=\int_{0}^{1}z^{a-1}{\left(1-z\right)}^{b-1}dz$ and τi,j,l,q=Ki,j,l(δ(l+1)−1q). Furthermore, the *m*th central moment of X is given by

μr′=E(x−μ1′)r=∑k=0r(−1)krkμ1′kμr−k′.

Table 1 gives some different statistical measures such as mean $\left(\mu_{1}^{\prime }\right)$, variance $\left(\sigma^{2}\right)$, skewness (SK), and kurtosis (KU) for some values of parameters.

From Table 1, we conclude that the NEITL distribution is skewed to the right and leptokurtic.

### 3.2. Incomplete Moments

The *r*th incomplete moment, say, $\eta_{r}\left(y\right)$ of *X*, is obtained from (11) as follows:

(14) $$\begin{array}{cc} \eta_{r}\left(y\right) & =2\delta \sum_{i,j=0}^{\infty }\sum_{l=0}^{u+1}K_{i,j,l}\;\int_{0}^{y}\frac{x^{r+1}}{{\left(1+x\right)}^{2\delta (l+1)+2}}{\left(1+\frac{x}{1+x}\right)}^{\delta (l+1)-1}dx\\ & =2\delta \sum_{i,j,q=0}^{\infty }\sum_{l=0}^{u+1}\tau_{i,j,l,q}\;\beta \left(r+q+2,\delta \left(l+1\right)-r,\frac{y}{1+y}\right), \end{array}$$

where β(.,.t) stands for an incomplete beta function. The first incomplete moment, for r=1 in (14), is obtained. The famous applications of the first incomplete moment are the Lorenz and Bonferroni curves which are defined, respectively, by $Lz\left(m\right)=\frac{\eta_{1}\left(m\right)}{\mu_{1}^{\prime }}$ and $Bu\left(m\right)=\frac{Lz(m)}{F(m)}$.

### 3.3. Quantile Function

The quantile function of the NEITL distribution, say, $x=Q\left(x\right)=F^{-1}\left(u\right)$, is derived by inverting (5) as follows:

(15) $$x=\sqrt{v^{2}-v}-v,$$

where $v=1-{\left[1-\sqrt{\frac{{\left(1-u\right)}^{\frac{1}{\theta }}-1}{\left(1-\theta \right){\left(1-u\right)}^{\frac{1}{\theta }}-1}}\;\right]}^{\frac{-1}{\delta }}$.

In particular, the first quartile, say, $Q_{1}$; the second quartile, say, $Q_{2}$; and the third quartile, say, $Q_{3}$ are obtained by setting x = 0.25, 0.5, and 0.75, respectively, in (15). The Bowley’s skewness depends on quartiles as follows:

(16) $$SK=\frac{Q(0.75)-2Q(0.5)+Q(0.25)}{Q(0.75)-Q(0.5)},$$

where *Q*(.) is the NEITL quantile function. The Moor’s kurtosis is given as

(17) $$KU=\frac{Q(0.875)-Q(0.625)-Q(0.375)+Q(0.125)}{Q(0.75)-Q(0.25)}.$$

**Figure 2:**
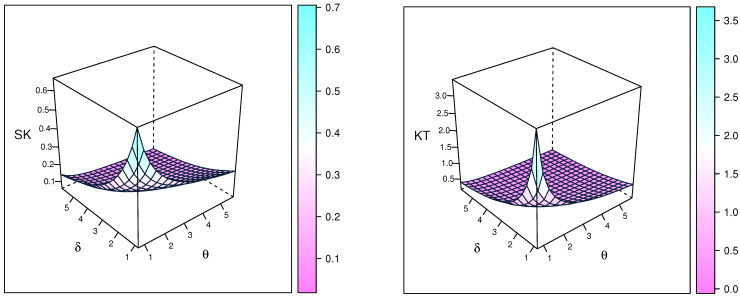
Plots of the skewness and kurtosis of the NEITL distribution.

Skewness and kurtosis plots of the NEITL distribution, based on quantiles, are exhibited in Figure 2.

### 3.4. Rényi and Other Entropies

Here, we obtain Rényi and ρ-entropies. The Rényi entropy, Ξ(b), of a random variable *X*, is defined by

(18) $$\Xi \left(b\right)=\frac{1}{1-b}\log\left[\int_{0}^{\infty }f^{b}\left(x\right)dx\right],$$

where b>0 and b≠0. Using expansions in (6) and after some simplification, then $f^{b}\left(x\right)$ should be written as:

(19) fb(x)=∑i,j=0∞(−1)jb(θ+1)+i−1b(θ+1)−1b(v)j(1−θ)i(4θ2δx)b(1+2x)b(δ−1)(1+x)b(2δ+1)1−(1+2x)δ(1+x)2δb+2(j+i).

Again, using the binomial expansions more than one times leads to

(20) $$f^{b}\left(x\right)=\sum_{i,j,k,m=0}^{\infty }\Psi_{i,j,k,m}x^{b+m}{\left(1+x\right)}^{-\delta k-b\delta -2b-m},$$

where Ψi,j,k,m=(−1)j+kb(θ+1)+i−1b(θ+1)−1b(v)jb+ukδk+b(δ−1)m(1−θ)i(4θ2δ)b. Substituting (20) in (18) gives

(21) $$\begin{array}{cc} \Xi (b) & =\frac{1}{1-b}\log\left[\int_{0}^{\infty }\sum_{i,j,k,m=0}^{\infty }\Psi_{i,j,k,m}x^{b+m}{\left(1+x\right)}^{-\delta k-b(\delta +2)-m}dx\right]\\ & =\frac{1}{1-b}\log\left[\sum_{i,j,k,m=0}^{\infty }\Psi_{i,j,k,m}\beta (b+m+1,\delta k+b(\delta +1)-1)\right]. \end{array}$$

The ρ entropy, E(ρ), is defined as follows:

(22) $$E\left(\rho \right)=\frac{1}{\rho -1}\log\left[1-\int_{0}^{\infty }f^{\rho }\left(x\right)dx\right];\;\rho >0\;and\;\rho \neq 0.$$

The ρ entropy of the NEITL takes the form

(23) $$E\left(\rho \right)=\frac{1}{\rho -1}\log\left[1-\sum_{i,j,k,m=0}^{\infty }\Psi_{i,j,k,m}\beta (b+m+1,\delta k+b(\delta +1)-1)\right].$$

## 4. Reliability Analysis

In this section, we discus the reliability analysis in terms of hazard, survival, and S–S reliability for the NEITL distribution.

### 4.1. Hazard and Survival Reliability

The survival function (SF) of the NEITL distribution is given by

(24) $$SF\left(x;\Omega \right)={\left[\frac{1-{\left[1-\frac{{\left(1+2x\right)}^{\delta }}{{\left(1+x\right)}^{2\delta }}\right]}^{2}}{1-\left(1-\theta \right){\left[1-\frac{{\left(1+2x\right)}^{\delta }}{{\left(1+x\right)}^{2\delta }}\right]}^{2}}\right]}^{\theta };\;x>0,\;\theta ,\delta >0.$$

**Figure 3:**
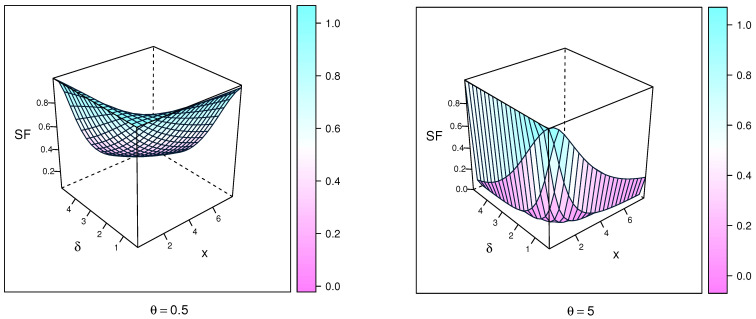
SF plots of the NEITL distribution.

Figure 3 gives SF plots of the NEITL distribution for specific values of parameters.

The HF of the NEITL distribution is given by

(25) $$HF\left(x;\Omega \right)=4\theta^{2}\delta x\frac{{\left(1+2x\right)}^{\delta -1}}{{\left(1+x\right)}^{2\delta +1}}\left[1-\frac{{\left(1+2x\right)}^{\delta }}{{\left(1+x\right)}^{2\delta }}\right]\frac{{\left(1-{\left[1-\frac{{\left(1+2x\right)}^{\delta }}{{\left(1+x\right)}^{2\delta }}\right]}^{2}\right)}^{-1}}{1-\left(1-\theta \right){\left[1-\frac{{\left(1+2x\right)}^{\delta }}{{\left(1+x\right)}^{2\delta }}\right]}^{2}}.$$

**Figure 4:**
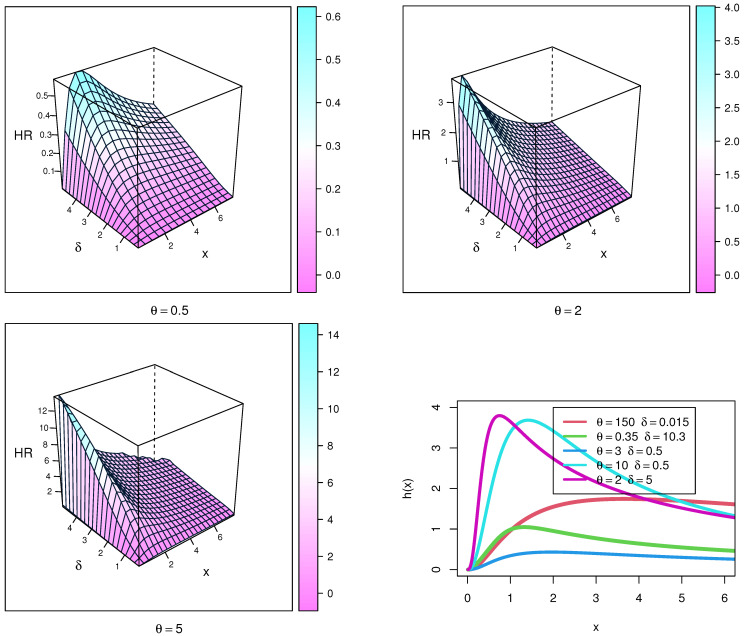
HF plots of the NEITL distribution.

The HF plots of the NEITL distribution are displayed in Figure 4 to control sequence for certain values of parameters. These figures show that the HF of the NEITL distribution can be increasing, decreasing, or upside-down shaped.

### 4.2. Stress–Strength Reliability

**Figure 5:**
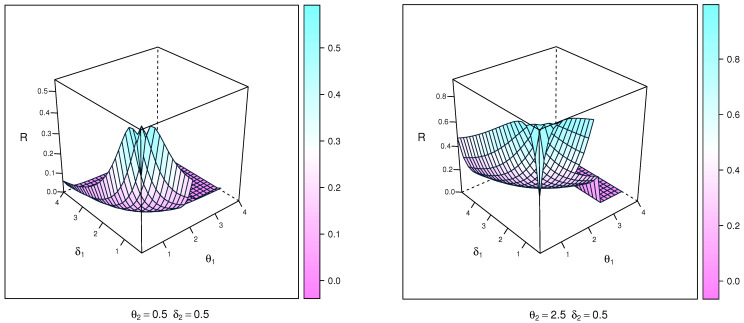

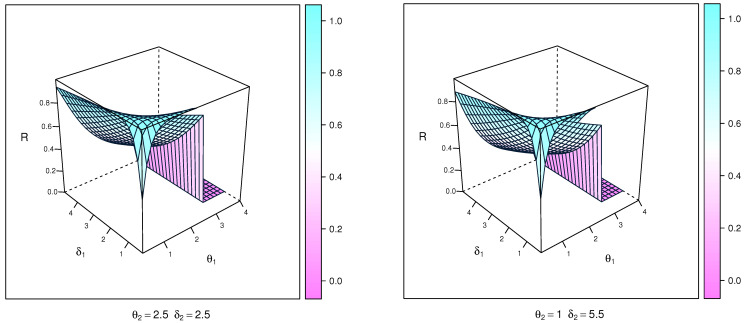
Stress–strength plots of the NEITL distribution.

The stress–strength model is extensively used in reliability estimation. The S–S model has many applications in physics and engineering, including strength failure testing, structural modeling, estimating the deterioration of rocket motors, and modeling the static fatigue of ceramic components. In the S–S model, reliability *R* measures the reliability of the component that has strength $X_{1}$ when it is subjected to random stress $X_{2}$. The component fails if the applied stress exceeds its strength: $R=P(X_{2}<X_{1})$. For more information about this model, see Abu El Azm et al. [17], Sabry et al. [18], Yousef and Almetwally [19], and Hassan et al. [20]. Let $X_{1}$ and $X_{2}$ be two independent random variables with NEITL$\left(\delta_{1},\theta \right)$ and NEITL$\left(\delta_{2},\theta \right)$ distributions, respectively. Hence, the S–S reliability is obtained, using the same expansions in (9) and (11) with different indicators, as follows:

(26) $$\begin{array}{cc} R & =1-A^{*}4\theta^{2}\delta_{1}\int_{0}^{\infty }x\frac{{\left(1+2x\right)}^{\delta_{1}\left(l_{1}+1\right)+l_{2}\delta_{2}-1}}{{\left(1+x\right)}^{2\delta_{1}\left(l_{1}+1\right)+2l_{2}\delta_{2}+1}}dx\\ \; & =1-A^{*}\frac{2\theta^{2}\delta_{1}}{\delta_{1}\left(l_{1}+1\right)+\delta_{2}l_{2}}\int_{0}^{\infty }2\left(\delta_{1}\left(l_{1}+1\right)+\delta_{2}l_{2}\right)x\frac{{\left(1+2x\right)}^{\delta_{1}\left(l_{1}+1\right)+l_{2}\delta_{2}-1}}{{\left(1+x\right)}^{2\delta_{1}\left(l_{1}+1\right)+2l_{2}\delta_{2}+1}}dx\\ \; & =1-A^{*}\frac{2\theta^{2}\delta_{1}}{\delta_{1}\left(l_{1}+1\right)+\delta_{2}l_{2}}, \end{array}$$

where A*=C1C2u2l2(−1)j2+j1+l2+l1(1−θ)i2+i2, C1=∑i1,j1=0∞∑l1=0u1+1i1+θθvj1u1+1l1, C2=∑i2,j2=0∞∑l2=0u2i2+vvθj2, $u_{1}=2\left(i_{1}+j_{1}\right)$ and $u_{2}=2\left(i_{2}+j_{2}\right)$. Plots of S–S model for some values of parameters are given in Figure 5.

## 5. Parameter Estimation

In this section, we use different point estimation methods to estimate the unknown parameters of the NEITL distribution. We use classical (ML and MPS) and non-classical (Bayesian) methods. In the last few years, parameter estimation using different methods has received great attention from many authors, such as Haj Ahmad and Almetwally [21], Basheer et al. [22], and Almetwally [15].

### 5.1. Maximum Likelihood Method

Let $x_{1},\cdots ,x_{n}$ be a random sample from the NEITL distribution with parameters θ and δ. The log-likelihood function of the NEITL can be written as:

(27) $$l\left(\Omega \right)=4^{n}\theta^{2n}\delta^{n}\prod_{i=1}^{n}\left[x_{i}\frac{{\left(1+2x_{i}\right)}^{\delta -1}}{{\left(1+x_{i}\right)}^{2\delta +1}}\left[1-A_{i}\left(\delta \right)\right]\frac{{\left(1-{\left[1-A_{i}\left(\delta \right)\right]}^{2}\right)}^{\theta -1}}{{\left(1-\left(1-\theta \right){\left[1-A_{i}\left(\delta \right)\right]}^{2}\right)}^{\theta +1}}\right],$$

where $A_{i}\left(\delta \right)=\frac{{\left(1+2x_{i}\right)}^{\delta }}{{\left(1+x_{i}\right)}^{2\delta }}$. The log-likelihood function of the NEITL distribution is

(28) $$\begin{array}{cc} \ell (\Omega )= & n\left(ln(4)+2ln(\theta )+ln(\delta )\right)+\sum_{i=1}^{n}ln\left(x_{i}\right)+\left(\delta -1\right)\sum_{i=1}^{n}ln\left(1+2x_{i}\right)+\sum_{i=1}^{n}ln\left[1-A_{i}\left(\delta \right)\right]-\\ \; & \left(2\delta +1\right)\sum_{i=1}^{n}ln\left(1+x_{i}\right)+\left(\theta -1\right)\sum_{i=1}^{n}ln\left(1-{\left[1-A_{i}\left(\delta \right)\right]}^{2}\right)-\left(\theta +1\right)\sum_{i=1}^{n}ln\left(1-\left(1-\theta \right){\left[1-A_{i}\left(\delta \right)\right]}^{2}\right). \end{array}$$

The ML estimators are obtained by solving the following equations:

$$\begin{array}{cc} \frac{\partial \ell (\Omega )}{\partial \theta }= & \frac{2n}{\theta }+\sum_{i=1}^{n}ln\left(1-{\left[1-A_{i}\left(\delta \right)\right]}^{2}\right)-\sum_{i=1}^{n}ln\left(1-\left(1-\theta \right){\left[1-A_{i}\left(\delta \right)\right]}^{2}\right)-\\ \; & \left(\theta +1\right)\sum_{i=1}^{n}\frac{{\left[1-A_{i}\left(\delta \right)\right]}^{2}}{1-\left(1-\theta \right){\left[1-A_{i}\left(\delta \right)\right]}^{2}}, \end{array}$$

and

$$\begin{array}{cc} \frac{\partial \ell (\Omega )}{\partial \delta }= & \frac{n}{\delta }+\sum_{i=1}^{n}ln\left(1+2x_{i}\right)+\left(v\right)\sum_{i=1}^{n}\frac{2\left[1-A_{i}\left(\delta \right)\right]A_{i}\left(\delta \right)ln\left[\frac{\left(1+2x_{i}\right)}{{\left(1+x_{i}\right)}^{2}}\right]}{1-{\left[1-A_{i}\left(\delta \right)\right]}^{2}}-2\sum_{i=1}^{n}ln\left(1+x_{i}\right)\\ \; & +\sum_{i=1}^{n}\frac{A_{i}\left(\delta \right)ln\left[\frac{\left(1+2x_{i}\right)}{{\left(1+x_{i}\right)}^{2}}\right]}{1-A_{i}\left(\delta \right)}-2\left(1-\theta \right)\left(\theta +1\right)\sum_{i=1}^{n}ln\frac{\left[1-A_{i}\left(\delta \right)\right]A_{i}\left(\delta \right)ln\left[\frac{\left(1+2x_{i}\right)}{{\left(1+x_{i}\right)}^{2}}\right]}{1-\left(1-\theta \right){\left[1-A_{i}\left(\delta \right)\right]}^{2}}. \end{array}$$

These equations cannot be solved explicitly; hence, a nonlinear optimization algorithm such as the Newton Raphson method is used.

### 5.2. Maximum Product Spacing

According to Cheng and Amin [23], the maximum product spacing method is an efficient estimation method that has proved to have some advantages with respect to other point estimation methods. Thus, we use MPS in this section to have point estimation of the unknown parameters of the NEITL distribution. This can be obtained by solving equations resulted from taking partial derivatives of logarithm of product spacing function G(Ω) which is written as:

$$G\left(\Omega \right)=\prod_{i=1}^{n+1}{\left({\left[\frac{1-{\left[1-A_{i-1}\left(\delta \right)\right]}^{2}}{1-\left(1-\theta \right){\left[1-A_{i-1}\left(\delta \right)\right]}^{2}}\right]}^{\theta }-{\left[\frac{1-{\left[1-A_{i}\left(\delta \right)\right]}^{2}}{1-\left(1-\theta \right){\left[1-A_{i}\left(\delta \right)\right]}^{2}}\right]}^{\theta }\right)}^{\frac{1}{n+1}},$$

and the logarithmic function of G(Ω)

(29) $$\log G\left(\Omega \right)=\frac{1}{n+1}\sum_{i=1}^{n+1}\ln\left({\left[\frac{1-{\left[1-A_{i-1}\left(\delta \right)\right]}^{2}}{1-\left(1-\theta \right){\left[1-A_{i-1}\left(\delta \right)\right]}^{2}}\right]}^{\theta }-{\left[\frac{1-{\left[1-A_{i}\left(\delta \right)\right]}^{2}}{1-\left(1-\theta \right){\left[1-A_{i}\left(\delta \right)\right]}^{2}}\right]}^{\theta }\right).$$

The MPS estimators of Ω are obtained by differentiating the log-product equation (Equation (29)) with respect to each parameter separately. We can solve the nonlinear system of equations by using any iterative technique, such as conjugate-gradient algorithms. Over the last few years, the estimation parameters of such models have been improved under censoring schemes—for instance, by Almetwally et al. [24] and El-Sherpieny et al. [25].

### 5.3. Bayesian Estimation

Bayesian method provide statistical inferences that are based on the prior distribution and loss function that are chosen. All parameters are treated as random variables with certain distributions, termed the prior distribution in this method. We must choose one if prior information is not available, which is frequently the case. The independent gamma distributions are our priors of choice because prior distribution selection plays such an essential role in parameter estimation. The joint prior distribution can be written as follows:

(30) $$\pi \left(\Omega \right)\propto \theta^{a_{1}-1}\delta^{a_{2}-1}e^{-(b_{1}\theta +b_{2}\delta )}.$$

The joint posterior density function of Ω is obtained from (27) and (30):

(31) $$\pi \left(\Omega |\underline{x}\right)=\frac{\ell \left(\underline{x}|\Omega \right).\pi \left(\Omega \right)}{\int_{\Omega }^{}\ell \left(\underline{x}|\Omega \right).\pi \left(\Omega \right)d\Omega },$$

where $\ell \left(\underline{x}|\Omega \right)\propto \theta^{2n}\delta^{2n}\prod_{i=1}^{n}\frac{{\left(1+2x_{i}\right)}^{\delta -1}}{{\left(1+x_{i}\right)}^{2\delta +1}}\left[1-A_{i}\left(\delta \right)\right]\frac{{\left(1-{\left[1-A_{i}\left(\delta \right)\right]}^{2}\right)}^{\theta -1}}{{\left(1-\left(1-\theta \right){\left[1-A_{i}\left(\delta \right)\right]}^{2}\right)}^{\theta +1}}$. Then, the posterior NEITL distribution is

(32) $$\pi \left(\Omega |\underline{x}\right)\propto \theta^{2n+a_{1}-1}\delta^{2n+a_{2}-1}e^{-(b_{1}\theta +b_{2}\delta )}\prod_{i=1}^{n}\frac{{\left(1+2x_{i}\right)}^{\delta -1}}{{\left(1+x_{i}\right)}^{2\delta +1}}\left[1-\frac{{\left(1+2x_{i}\right)}^{\delta }}{{\left(1+x_{i}\right)}^{2\delta }}\right]\frac{{\left(1-{\left[1-A_{i}\left(\delta \right)\right]}^{2}\right)}^{\theta -1}}{{\left(1-\left(1-\theta \right){\left[1-A_{i}\left(\delta \right)\right]}^{2}\right)}^{\theta +1}}.$$

The conditional posterior distribution is as follows:

(33) $$\pi \left(\theta |\delta ,\underline{x}\right)\propto \theta^{2n+a_{1}-1}e^{-b_{1}\theta }\prod_{i=1}^{n}\frac{{\left(1-{\left[1-A_{i}\left(\delta \right)\right]}^{2}\right)}^{\theta -1}}{{\left(1-\left(1-\theta \right){\left[1-A_{i}\left(\delta \right)\right]}^{2}\right)}^{\theta +1}},$$

and

(34) $$\pi \left(\delta |\theta ,\underline{x}\right)\propto \delta^{2n+a_{2}-1}e^{-b_{2}\delta }\prod_{i=1}^{n}\frac{{\left(1+2x_{i}\right)}^{\delta -1}}{{\left(1+x_{i}\right)}^{2\delta +1}}\left[1-A_{i}\left(\delta \right)\right]\frac{{\left(1-{\left[1-A_{i}\left(\delta \right)\right]}^{2}\right)}^{\theta -1}}{{\left(1-\left(1-\theta \right){\left[1-A_{i}\left(\delta \right)\right]}^{2}\right)}^{\theta +1}}.$$

The loss function, on the other hand, is crucial in Bayesian approaches. The symmetric and asymmetric loss functions are used to create the majority of Bayesian inference processes. The Bayes estimators of Ω, say, $\left({\hat{\theta }}_{B},{\hat{\delta }}_{B}\right)$, based on a squared error loss function, are given by

(35) $$\begin{array}{cc} {\hat{p}}_{B-SEL}\left(\theta ,\delta \right) & =E_{\left(\theta ,\delta \underline{x}\right)}\left[p\left(\theta ,\delta \right)\right]\\ & =\int_{0}^{\infty }\int_{0}^{\infty }p\left(\theta ,\delta \right)\times \pi \left(\Omega |\underline{x}\right)d\theta d\delta . \end{array}$$

It is noted that the integrals given by (35) cannot be obtained explicitly. Due to that, we used the Markov chain Monte Carlo technique (MCMC) to find approximate values of integrals (35). Many studies have used the MCMC technique, such as El-Sherpieny et al. [26], Almongy et al. [27], Haj Ahmad et al. [28], Bantan et al. [29], Almetwally et al. [24], Al-Omari et al. [30], Al-Babtain et al. [31], and Hassan and Zaki [32].

## 6. Simulation

A simulation study has been conducted to examine the performances of point estimates in terms of their average estimates (AE), mean squared errors (MSE), interval estimates, and lengths of confidence interval (L.CI). The simulation study was carried out with various parameter values and sample sizes. This section is divided into two parts.

For the first reliability analysis: The parameters of the NEITL distribution were (θ;δ) = (0.5; 0.5) and (0.5; 3) for the results in Table 2 and (θ;δ) = (3; 0.5) and (3; 3) for the results in Table 3. The sample sizes were n = 30, 80, and 150, respectively. We selected time (Q) to determine the HF and SF of the NEITL distribution where $R_{1}=SF\left(Q=0.25;\hat{\Omega }\right)$, $R_{2}=SF\left(Q=0.35;\hat{\Omega }\right)$, $H_{1}=HF\left(Q=0.25;\hat{\Omega }\right)$, and $H_{2}=HF\left(Q=0.35;\hat{\Omega }\right)$. The various simulation results are based on a total of 10,000 repetitions. The Bayes estimates are based on 10,000 samples and were derived using the MCMC approach. In Table 2 and Table 3, the AE, MSEs, and L.CI of the various approaches are displayed.

Secondly, we estimated the reliability of the S–S model. The parameters of the NEITL distribution were $\left(\theta_{1};\delta_{1};\theta_{2};\delta_{2}\right)$ = (0.6; 0.75; 0.65; 2.5) is case 1 and (0.6; 0.75; 2.65; 2.5)—see Table 4; and $\left(\theta_{1};\delta_{1};\theta_{2};\delta_{2}\right)$ = (2; 1.75; 2.5; 2.5) is case 3 and (0.6; 2.75; 2.65; 2.5) is case 4—see Table 5. The sample sizes of S–S model were (n, m) = (25, 30), (80, 70), and (150, 120), respectively.

Table 2, Table 3, Table 4 and Table 5 present the results, which highlight some interesting facts. As the sample size gets larger, the estimates get more accurate, demonstrating that they are asymptotically unbiased. Furthermore, the MSE decreases as the sample size increases in all cases, demonstrating that the various estimates are consistent. When comparing the various estimates, we can observe that in the majority of cases, the Bayes estimates have the lowest MSE. MPS estimate is a good alternative for ML estimate (MLE). The L-CI for the estimates approach zero as the sample size (n) increases, indicating that the CI for the largest sample size is the shortest CI. The greater the time we tested (Q), the lower the HF and SF values. When estimating the reliability of the S–S model in most cases, we received large values close to one, which indicates the quality of the model used.

## 7. Application of Real Data

To demonstrate the NEITL model’s flexibility and applicability in practice, two real life datasets are analyzed in this section. The NEITL distribution is compared to the ITL, exponentiated Lomax (EL), exponentiated exponential (ExEx), Weibull (W), Kumaraswamy Weibull (KW), modified Kies ITL (MKITL), and odd Weibull ITL (OWITL) distributions for the first batch of data. The NEITL distribution is compared to the ITL, EL, ExEx, W, KW, Kumaraswamy ITL (KIT), MKITL, and OWITL distributions for the second dataset. The approach of maximum likelihood was used to estimate the unknown parameters of the specified models for the two real datasets. To compare all of the models, the following statistics are used: Kolmogorov–Smirnov (KS), Cramer–von Mises (CVM), Anderson–Darling (AD), Akaike information criterion (AIC), and Bayesian information criterion (BIC).

### 7.1. Survival Times

Bjerkedal [33] observed and reported the survival periods (in days) of 72 guinea pigs infected with virulent tubercle bacilli in the first dataset. These data are as follows: 0.1, 0.33, 0.44, 0.56, 0.59, 0.59, 0.72, 0.74, 0.92, 0.93, 0.96, 1, 1, 1.02, 1.05, 1.07, 1.07, 1.08, 1.08, 1.08, 1.09, 1.12, 1.13, 1.15, 1.16, 1.2, 1.21, 1.22, 1.22, 1.24, 1.3, 1.34, 1.36, 1.39, 1.44, 1.46, 1.53, 1.59, 1.6, 1.63, 1.63, 1.68, 1.71, 1.72, 1.76, 1.83, 1.95, 1.96, 1.97, 2.02, 2.13, 2.15, 2.16, 2.22, 2.3, 2.31, 2.4, 2.45, 2.51, 2.53, 2.54, 2.54, 2.78, 2.93, 3.27, 3.42, 3.47, 3.61, 4.02, 4.32, 4.58, and 5.55.

MLEs, SE, KS, CVM, AD, AIC, and BIC values for the first dataset are summarized in Table 6. The NEITL model has the least values for the statistical measures among all fitted models, as shown in the table.

**Figure 6:**
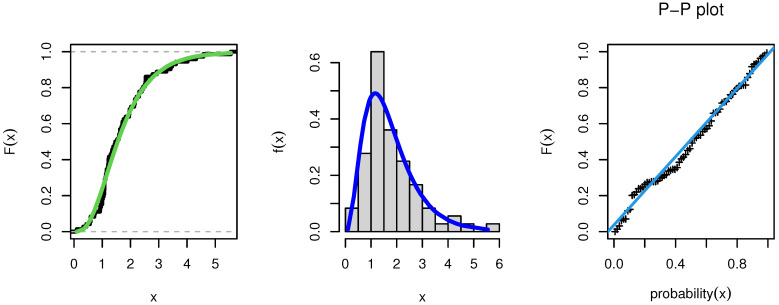
PDF, CDF, and PP plot of the NEITL distribution:Survival Times.

As a result, the NEITL model might be the best option. Figure 6 shows the estimated CDF, estimated PDF, and PP plot of the fitted NEITL model, respectively.

### 7.2. Example of Reliability of the S–S Model

Nelson [34] (Ch. 10, Table 4.1) calculated the time it takes for an insulating fluid to break down under high voltage stress in minutes. The failure times were observed in groups of ten insulating fluids, with each group reporting data on ten of them. Consider the following two sets of failure time data samples presented as follows for the purpose of showing the methods of inference outlined in the preceding sections:

 Group 1: 0.31, 0.66, 1.54, 1.70, 1.82, 1.89, 2.17, 2.24, 4.03, and 9.99.
 Group 2: 0.49, 0.64, 0.82, 0.93, 1.08, 1.99, 2.06, 2.15, 2.57, and 4.75.

MLEs, SE, KS, CVM, AD, AIC, and BIC values for the data of Group 1 and Group 2 are summarized in Table 7 and Table 8. The NEITL model resulted in the best values for the statistical measures among all fitted models, as shown in theses tables. Table 9 provided MLE, MPS, and Bayesian estimates for reliability of the S–S model.

**Figure 7:**
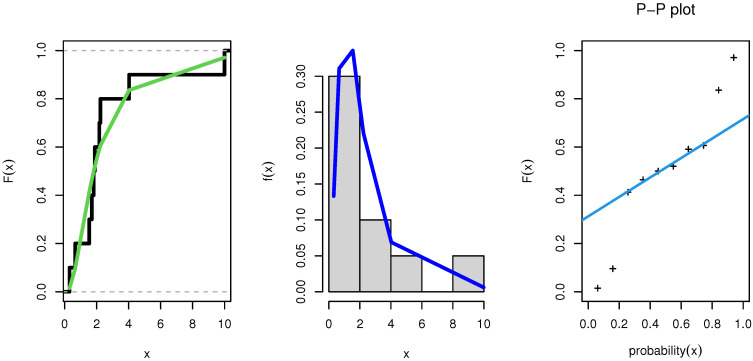
PDF, CDF and PP plot of the NEITL distribution: Group 1.

**Figure 8:**
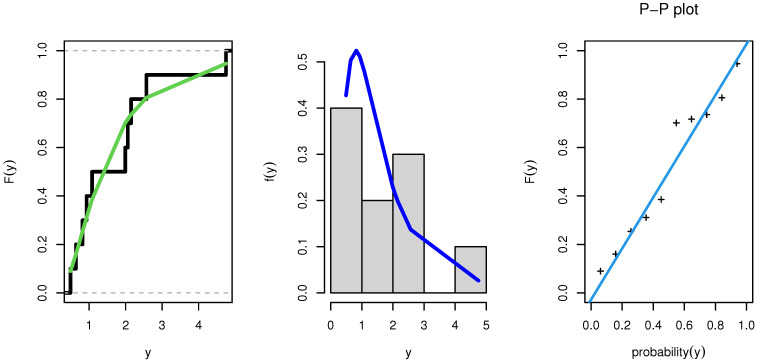
PDF, CDF, and PP plot of the NEITL distribution: Group 2.

As a result, the NEITL model might be the best option. Figure 7 and Figure 8 show the estimated CDFs, estimated PDFs, and PP plot of the fitted NEITL model, respectively.

**Figure 9:**
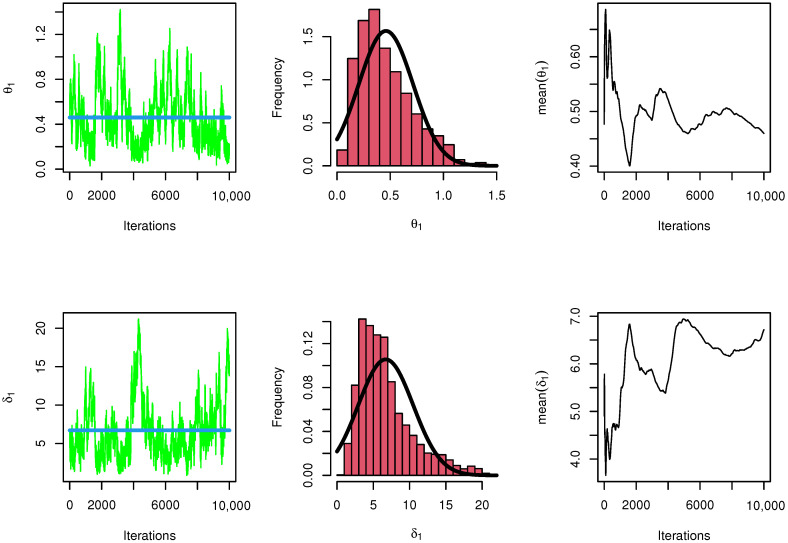
Trace, proposed distribution, and convergence of MCMC results for $\theta_{1},\delta_{1}$.

**Figure 10:**
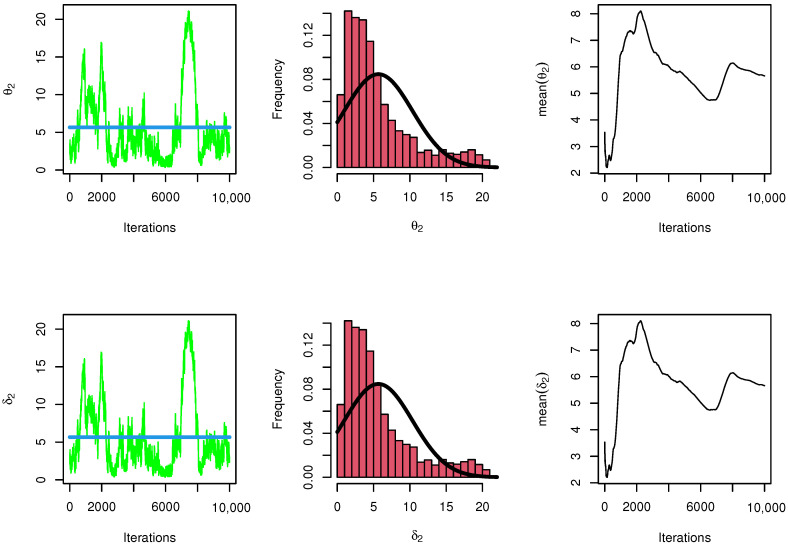
Trace, proposed distribution, and convergence of MCMC results for $\theta_{2},\delta_{2}$.

Figure 9 and Figure 10 show convergence plots of MCMC for parameter estimates of the NEITL distribution.

## 8. Conclusions

A new two-parameter lifetime model, named “new exponential inverted Topp–Leone”, is introduced in this paper. The new distribution gives more flexibility and wide applicability compared to the existing models. It appears that the shape of the distribution depends on the values of the parameters. Some of the novel hazard rates that can be used are: decreased, constant hazard rate, increasing hazard rate, upside down (reversed bathtub shape), and increasing-constant hazard rate. Several mathematical and distributional properties, such as ordinal moments, incomplete moments, quantile function, Renyi, and ρ entropies, were described in detail. The new density is a linear combination of the well-known inverted Topp–Leone density. The reliability of stress strength was calculated. Using Bayesian and non-Bayesian estimation methods, the parameters of the NEITL distribution were estimated. In simulation research, statistical analysis was used to compare these methods in order to evaluate their effectiveness and investigate how these estimates perform for different sample sizes and parameter values. The simulation results indicate that the Bayes estimate performed the best in the smaller MSE sense. In most cases, we received large values near to one when calculating the S–S model’s reliability, indicating that the model is of good quality. Furthermore, we propose using MPS estimation instead of ML estimation. To demonstrate the use of the novel distribution, two real-life datasets from the engineering and medical fields were studied. In addition, the use of these data in the stress–strength model has been validated. We hope that this distribution could be used in more areas.

## Figures and Tables

**Table 1:** Moments measures for NEITL distribution.

(δ,θ)	$\mu_{1}^{\prime }$	$\sigma^{2}$	SK	KU
(2,3)	0.739	0.204	2.624	23.372
(3,3)	0.534	0.083	1.871	11.412
(5,3)	0.369	0.032	1.409	7.371
(5,4)	0.293	0.016	1.07	5.572
(5,5)	0.248	0.01	0.847	4.602
(1,5)	0.787	0.192	2.089	17.382
(2,7)	0.414	0.048	1.599	8.225
(3,7)	0.295	0.018	1.222	6.201

**Table 2:** Accuracy measures for parameters of the NEITL distribution, and reliability analysis for different periods of time for θ=0.5.

θ=0.5				MLE			MPS			Bayesian		
δ	**n**			**AE**	**MSE**	**L.CI**	**AE**	**MSE**	**L.CI**	**AE**	**MSE**	**L.CI**
0.5	30		θ	0.0875	0.2002	1.7121	0.0813	0.2001	1.7009	0.0419	0.0635	0.8738
			δ	0.1646	0.2306	1.7411	0.0731	0.1905	1.6706	0.0400	0.0429	0.7872
		Q = 0.25	$R_{1}$	0.7500	0.0035	0.2262	0.7498	0.0032	0.2173	0.7359	0.0053	0.2714
			$H_{1}$	0.0043	1.29 × $10^{-6}$	0.0039	0.0041	9.11 × $10^{-7}$	0.0038	0.0042	1.49 × $10^{-6}$	0.0048
		Q = 0.35	$R_{2}$	0.6369	0.0051	0.2735	0.6489	0.0047	0.2651	0.6336	0.0078	0.3286
			$H_{2}$	0.0021	2.11 × $10^{-7}$	0.0016	0.0018	1.75 × $10^{-7}$	0.0017	0.0021	3.09 × $10^{-7}$	0.0021
	80		θ	0.1093	0.1784	1.6000	0.1621	0.1722	1.5607	0.0177	0.0215	0.5545
			δ	0.0822	0.1284	1.3681	0.0518	0.1230	1.2400	0.0243	0.0203	0.5598
		Q = 0.25	$R_{1}$	0.7458	0.0011	0.1265	0.7494	0.0010	0.1258	0.7415	0.0014	0.1317
			$H_{1}$	0.0040	3.51 × $10^{-7}$	0.0022	0.0039	3.13 × $10^{-7}$	0.0021	0.0040	3.84 × $10^{-7}$	0.0022
		Q = 0.35	$R_{2}$	0.6423	0.0016	0.1526	0.6472	0.0015	0.1516	0.6392	0.0021	0.1612
			$H_{2}$	0.0020	7.01 × $10^{-8}$	0.0010	0.0019	6.27 × $10^{-8}$	0.0010	0.0020	8.26 × $10^{-8}$	0.0010
	150		θ	0.1014	0.1627	1.5310	0.1412	0.1521	1.4619	0.0038	0.0068	0.3083
			δ	0.0627	0.0951	1.1839	0.0498	0.0910	1.1253	0.0103	0.0062	0.3008
		Q = 0.25	$R_{1}$	0.7472	0.0006	0.0966	0.7494	0.0006	0.0967	0.7469	6.11 × $10^{-4}$	0.0940
			$H_{1}$	0.0040	2.06 × $10^{-7}$	0.0016	0.0039	1.93 × $10^{-7}$	0.0016	0.0039	1.61 × $10^{-7}$	0.0016
		Q = 0.35	$R_{2}$	0.6440	0.0009	0.1147	0.6468	8.65 × $10^{-4}$	0.1146	0.6461	9.27 × $10^{-4}$	0.1181
			$H_{2}$	0.0020	4.39 × $10^{-8}$	0.0008	0.0019	4.07 × $10^{-8}$	0.0008	0.0019	3.49 × $10^{-8}$	0.0007
3	25		θ	0.0128	0.2007	1.7037	0.0140	0.0135	0.4524	0.0197	0.0086	0.3640
			δ	−0.0015	0.2298	1.7140	−0.0693	0.1306	1.3911	−0.0178	0.0681	1.0029
		Q = 0.25	$R_{1}$	0.7429	0.0032	0.2212	0.7535	0.0030	0.2148	0.7390	0.0037	0.2296
			$H_{1}$	0.2805	0.0043	0.2533	0.2671	0.0038	0.2412	0.2841	0.0049	0.2588
		Q = 0.35	$R_{2}$	0.6427	0.0047	0.2688	0.6562	0.0045	0.2625	0.6384	0.0054	0.2772
			$H_{2}$	0.2851	0.0036	0.2344	0.2722	0.0033	0.2234	0.2882	0.0042	0.2387
	80		θ	0.0233	0.0136	0.4482	0.0007	0.0029	0.2101	0.0067	0.0028	0.2001
			δ	−0.0012	0.1313	1.2926	−0.0189	0.0360	0.7405	−0.0068	0.0308	0.6843
		Q = 0.25	$R_{1}$	0.7475	0.0009	0.1203	0.7529	0.0009	0.1185	0.7466	0.0011	0.1252
			$H_{1}$	0.2740	0.0012	0.1339	0.2673	0.0011	0.1318	0.2745	0.0014	0.1406
		Q = 0.35	$R_{2}$	0.6473	0.0014	0.1464	0.6542	0.0014	0.1457	0.6464	0.0016	0.1538
			$H_{2}$	0.2791	0.0010	0.1257	0.2732	0.0010	0.1228	0.2800	0.0012	0.1319
	150		θ	0.0034	0.0035	0.2332	−0.0017	0.0018	0.1679	0.0032	0.0016	0.1496
			δ	0.0063	0.0746	1.0707	−0.0065	0.0224	0.5869	−0.0042	0.0150	0.4773
		Q = 0.25	$R_{1}$	0.7494	0.0006	0.0971	0.7528	0.0006	0.0961	0.7484	0.0007	0.0962
			$H_{1}$	0.2716	0.0008	0.1083	0.2675	0.0008	0.1069	0.2724	0.0009	0.1075
		Q = 0.35	$R_{2}$	0.6495	0.0009	0.1189	0.6538	0.0009	0.1182	0.6484	0.0010	0.1185
			$H_{2}$	0.2774	0.0007	0.1012	0.2736	0.0007	0.0996	0.2782	0.0008	0.1013

**Table 3:** Accuracy measures for parameters of the NEITL distribution, and reliability analysis for different periods of time for θ=3.

θ=3				MLE			MPS			Bayesian		
δ	**n**			**AE**	**MSE**	**L.CI**	**AE**	**MSE**	**L.CI**	**AE**	**MSE**	**L.CI**
0.5	30		θ	0.0044	0.4520	2.6372	−0.3054	0.4210	2.2450	−0.0197	0.0670	0.9916
			δ	0.0215	0.1230	1.3740	0.1181	0.0898	1.0801	0.0240	0.0115	0.3713
		Q = 0.25	$R_{1}$	0.7461	0.0038	0.2451	0.7479	0.0033	0.2276	0.7375	0.0046	0.2388
			$H_{1}$	0.4116	0.0115	0.4159	0.4015	0.0097	0.3852	0.4261	0.0140	0.4222
		Q = 0.35	$R_{2}$	0.6466	0.0056	0.2970	0.6508	0.0051	0.2775	0.6367	0.0065	0.2943
			$H_{2}$	0.4372	0.0112	0.4083	0.4220	0.0095	0.3776	0.4495	0.0138	0.4233
	80		θ	−0.0571	0.2683	2.0189	−0.1001	0.0945	1.1397	−0.0118	0.0279	0.6472
			δ	0.0404	0.0381	0.7487	0.0270	0.0103	0.3833	0.0098	0.0029	0.2039
		Q = 0.25	$R_{1}$	0.7470	0.0010	0.1241	0.7505	0.0009	0.1192	0.7455	0.0011	0.1232
			$H_{1}$	0.4056	0.0028	0.2067	0.3995	0.0027	0.2031	0.4099	0.0033	0.2120
		Q = 0.35	$R_{2}$	0.6472	0.0015	0.1515	0.6517	0.0014	0.1477	0.6449	0.0017	0.1532
			$H_{2}$	0.4296	0.0027	0.2046	0.4232	0.0027	0.2019	0.4345	0.0033	0.2125
	150		θ	0.0134	0.0735	1.0617	−0.0557	0.0382	0.7353	−0.0086	0.0117	0.4165
			δ	0.0038	0.0044	0.2605	0.0118	0.0028	0.2036	0.0040	0.0014	0.1387
		Q = 0.25	$R_{1}$	0.7493	0.0005	0.0885	0.7510	0.0005	0.0877	0.7486	0.0006	0.0927
			$H_{1}$	0.4029	0.0015	0.1520	0.3993	0.0015	0.1506	0.4042	0.0017	0.1597
		Q = 0.35	$R_{2}$	0.6494	0.0008	0.1103	0.6518	0.0008	0.1095	0.6486	0.0009	0.1161
			$H_{2}$	0.4279	0.0015	0.1521	0.4237	0.0015	0.1506	0.4290	0.0017	0.1601
3	25		θ	0.7823	3.2109	6.3225	−0.1464	0.3771	2.3388	−0.0035	0.0587	0.9477
			δ	0.0619	3.2238	7.0377	0.2793	0.6266	2.9050	−0.0072	0.0667	1.0296
		Q = 0.25	$R_{1}$	0.7490	0.0031	0.2172	0.7527	0.0026	0.1984	0.7515	0.0017	0.1578
			$H_{1}$	2.3694	0.3144	2.1952	2.3050	0.2756	2.0555	2.3288	0.1847	1.6417
		Q = 0.35	$R_{2}$	0.6495	0.0046	0.2665	0.6554	0.0040	0.2485	0.6528	0.0027	0.2000
			$H_{2}$	2.8692	0.4192	2.5313	2.7668	0.3667	2.3667	2.8072	0.2489	1.9051
	80		θ	0.3595	1.3131	4.2674	−0.0903	0.1382	1.4145	−0.0080	0.0269	0.6306
			δ	0.0021	1.2808	4.4386	0.1369	0.2231	1.7731	−0.0052	0.0276	0.6449
		Q = 0.25	$R_{1}$	0.7513	0.0010	0.1262	0.7519	0.0008	0.1126	0.7519	0.0007	0.0992
			$H_{1}$	2.3306	0.0959	1.2146	2.3116	0.0863	1.1484	2.3194	0.0739	1.0318
		Q = 0.35	$R_{2}$	0.6518	0.0016	0.1545	0.6532	0.0013	0.1420	0.6528	0.0011	0.1260
			$H_{2}$	2.8178	0.1231	1.3759	2.7823	0.1157	1.3265	2.7978	0.1002	1.2029
	150		θ	0.2715	0.9255	3.6196	−0.0411	0.0771	1.0772	−0.0064	0.0098	0.3843
			δ	0.0304	1.1950	4.2856	0.0627	0.1185	1.3276	−0.0006	0.0108	0.3920
		Q = 0.25	$R_{1}$	0.7514	0.0006	0.0982	0.7515	0.0004	0.0829	0.7510	0.0003	0.0653
			$H_{1}$	2.3255	0.0533	0.9045	2.3185	0.0465	0.8429	2.3267	0.0303	0.6728
		Q = 0.35	$R_{2}$	0.6519	0.0009	0.1180	0.6523	0.0007	0.1044	0.6514	0.0005	0.0825
			$H_{2}$	2.8103	0.0657	1.0047	2.7942	0.0620	0.9720	2.8067	0.0409	0.7801

**Table 4:** Accuracy measures for parameters of the NEITL distribution, and reliability analysis for different periods of time for case 1 and case 2.

Case			MLE			MPS			Bayesian		
	**n, m**		**AE**	**MSE**	**L.CI**	**AE**	**MSE**	**L.CI**	**AE**	**MSE**	**L.CI**
1	25, 30	$\theta_{1}$	0.0616	0.1473	1.4864	0.1520	0.1840	1.5742	0.0335	0.0437	0.7637
		$\delta_{1}$	0.1833	0.3414	2.1768	0.0562	0.2808	2.0675	0.0211	0.0481	0.8245
		$\theta_{2}$	0.0443	0.0724	1.0414	0.0726	0.0649	0.9579	0.0079	0.0134	0.4490
		$\delta_{2}$	0.1103	0.7321	3.3293	−0.1033	0.5550	2.8949	−0.0034	0.0622	0.9261
		R	0.8257	0.0008	0.1057	0.8188	0.0009	0.1048	0.8198	0.0026	0.1168
	80, 70	$\theta_{1}$	0.0510	0.1108	1.2910	0.0995	0.1361	1.3942	0.0248	0.0156	0.4902
		$\delta_{1}$	0.1207	0.2031	1.7038	0.0646	0.1908	1.6953	0.0008	0.0223	0.5705
		$\theta_{2}$	0.0097	0.0211	0.5689	0.0351	0.0307	0.6739	0.0091	0.0053	0.2729
		$\delta_{2}$	0.0776	0.2878	2.0828	−0.0305	0.3172	2.2068	−0.0074	0.0334	0.7208
		R	0.8316	0.0003	0.0692	0.8281	0.0003	0.0697	0.8300	0.0003	0.0694
	150, 120	$\theta_{1}$	0.0364	0.0790	1.0937	0.0613	0.0888	1.1441	0.0034	0.0051	0.2641
		$\delta_{1}$	0.0905	0.1390	1.4189	0.0609	0.1388	1.4424	0.0072	0.0071	0.3124
		$\theta_{2}$	0.0146	0.0185	0.5302	0.0266	0.0235	0.5929	0.0011	0.0024	0.1825
		$\delta_{2}$	0.0424	0.2233	1.8467	−0.0125	0.2486	1.9558	0.0088	0.0104	0.4007
		R	0.8333	0.0002	0.0528	0.8310	0.0002	0.0534	0.8319	0.0002	0.0459
2	25, 30	$\theta_{1}$	0.0439	0.1438	1.4778	0.1211	0.1711	1.5518	0.0366	0.0404	0.7013
		$\delta_{1}$	0.1944	0.3157	2.0686	0.0782	0.2577	1.9680	0.0241	0.0491	0.8161
		$\theta_{2}$	0.2202	0.5042	2.6489	−0.1283	0.2027	1.6934	−0.0028	0.0540	0.8770
		$\delta_{2}$	−0.0569	0.4444	2.6064	0.1952	0.3302	2.1209	0.0008	0.0512	0.8629
		R	0.9413	0.0006	0.0964	0.9350	0.0009	0.1055	0.9419	0.0014	0.0843
	80, 70	$\theta_{1}$	0.0483	0.1344	1.4775	0.1200	0.1689	1.5422	0.0326	0.0373	0.6871
		$\delta_{1}$	0.1839	0.3006	2.0270	0.0759	0.2500	1.9394	0.0248	0.0469	0.8096
		$\theta_{2}$	0.2154	0.4842	2.5963	−0.1161	0.1949	1.6714	−0.0023	0.0509	0.8596
		$\delta_{2}$	−0.0524	0.4238	2.5462	0.1866	0.3088	2.0538	0.0008	0.0469	0.8333
		R	0.9420	0.0006	0.0915	0.9362	0.0008	0.0996	0.9423	0.0013	0.0827
	150, 120	$\theta_{1}$	0.0203	0.0837	1.1326	0.0384	0.0833	1.1222	0.0276	0.0364	0.6734
		$\delta_{1}$	0.1220	0.1737	1.5636	0.0861	0.1475	1.4688	0.0382	0.0469	0.8254
		$\theta_{2}$	0.0302	0.0668	1.0074	−0.1569	0.2685	1.9378	0.0046	0.0487	0.8573
		$\delta_{2}$	−0.0076	0.0753	1.0761	0.2513	0.4349	2.3923	−0.0051	0.0533	0.8637
		R	0.9456	0.0001	0.0351	0.9436	0.0001	0.0364	0.9495	0.0001	0.0314

**Table 5:** Accuracy measures for parameters of the NEITL distribution, and reliability analysis for different periods of time for case 3 and case 4.

Case			MLE			MPS			Bayesian		
	**n, m**		**AE**	**MSE**	**L.CI**	**AE**	**MSE**	**L.CI**	**AE**	**MSE**	**L.CI**
3	25, 30	$\theta_{1}$	0.7656	3.1827	6.3231	0.0110	0.7518	3.4021	0.0212	0.0454	0.8269
		$\delta_{1}$	0.1439	1.7960	5.2283	0.2880	0.7248	3.1438	−0.0114	0.0521	0.8734
		$\theta_{2}$	0.9499	4.4971	7.4397	−0.0677	0.5883	2.9978	0.0097	0.0520	0.8664
		$\delta_{2}$	0.1369	3.2851	7.0917	0.3426	1.0036	3.6939	−0.0015	0.0648	0.9911
		R	0.7228	0.0037	0.2406	0.7202	0.0036	0.2337	0.7198	0.0034	0.2146
	80, 70	$\theta_{1}$	0.3765	1.3337	4.2840	0.0197	0.3368	2.2759	0.0025	0.0171	0.4865
		$\delta_{1}$	0.0930	0.9049	3.7147	0.1322	0.3505	2.2643	0.0035	0.0202	0.5197
		$\theta_{2}$	0.3805	1.4584	4.4973	−0.0547	0.3464	2.2996	0.0025	0.0249	0.6042
		$\delta_{2}$	0.0725	1.2650	4.4042	0.2302	0.5551	2.7805	−0.0022	0.0294	0.6962
		R	0.7256	0.0013	0.1402	0.7219	0.0012	0.1354	0.7202	0.0013	0.1402
	150, 120	$\theta_{1}$	0.2757	0.9253	3.6162	0.0430	0.2072	1.7780	0.0036	0.0088	0.3603
		$\delta_{1}$	0.0914	0.7308	3.3352	0.0487	0.1931	1.7136	−0.0055	0.0083	0.3559
		$\theta_{2}$	0.3190	1.1487	4.0149	−0.0215	0.2164	1.8236	0.0057	0.0095	0.3781
		$\delta_{2}$	0.0404	1.0387	3.9961	0.1224	0.3149	2.1490	−0.0092	0.0106	0.4029
		R	0.7243	0.0008	0.1076	0.7217	0.0007	0.1031	0.7218	0.0006	0.0964
4	25, 30	$\theta_{1}$	0.0873	0.1165	1.2946	0.1136	0.1002	1.1594	0.0204	0.0163	0.4498
		$\delta_{1}$	0.0713	1.1009	4.1077	−0.1877	0.8136	3.4619	−0.0133	0.0745	0.9809
		$\theta_{2}$	0.5909	2.0654	5.1406	−0.0731	0.4676	2.6679	−0.0043	0.0520	0.8618
		$\delta_{2}$	−0.0785	1.3511	4.5506	0.2558	0.6836	3.0852	0.0041	0.0580	0.9599
		R	0.9248	0.0005	0.0878	0.9246	0.0005	0.0888	0.9207	0.0009	0.0962
	80, 70	$\theta_{1}$	0.0788	0.0696	0.9878	0.0535	0.0363	0.7175	0.0081	0.0040	0.2443
		$\delta_{1}$	−0.0753	0.6112	3.0534	−0.1039	0.3955	2.4339	−0.0091	0.0302	0.6873
		$\theta_{2}$	0.2045	0.5337	2.7519	−0.0618	0.2220	1.8330	−0.0037	0.0227	0.5760
		$\delta_{2}$	−0.0210	0.4744	2.7013	0.1593	0.3110	2.0972	−0.0036	0.0235	0.5869
		R	0.9266	0.0002	0.0554	0.9259	0.0002	0.0557	0.9245	0.0002	0.0552
	150, 120	$\theta_{1}$	0.0477	0.0317	0.6736	0.0271	0.0158	0.4820	0.0040	0.0020	0.1743
		$\delta_{1}$	−0.0626	0.3421	2.2817	−0.0505	0.2179	1.8211	0.0010	0.0113	0.4141
		$\theta_{2}$	0.0683	0.2062	1.7614	−0.0761	0.1350	1.4107	0.0043	0.0095	0.3757
		$\delta_{2}$	0.0123	0.2282	1.8738	0.1348	0.1905	1.6290	−0.0017	0.0111	0.4077
		R	0.9260	0.0001	0.0419	0.9257	0.0001	0.0421	0.9258	0.0001	0.0395

**Table 6:** MLE with SE and other metrics: Survival Times.

		Estimation	SE	KS	CVM	AD	AIC	BIC
ITL	δ	2.0225	0.2384	0.2989	0.0942	0.6663	229.6917	231.9684
NEITL	θ	60.9983	19.8002	0.0902	0.0776	0.4946	193.1635	197.7168
	δ	0.0299	0.0549					
EL	α	3.7415	0.8152	0.0978	0.0766	0.4949	195.2402	202.0702
	β	37.0309	60.7818					
	λ	31.2893	54.2759					
ExEx	α	15.4717	20.7674	0.2194	0.2209	1.2910	210.8807	215.4340
	β	0.0240	0.0334					
W	α	1.8173	0.1583	0.7439	0.0865	0.5852	195.8812	200.4345
	β	0.2856	0.0544					
KW	α	0.7474	0.6138	0.0917	0.0878	0.5351	196.6326	205.7393
	β	0.9899	1.0882					
	λ	3.0474	3.9283					
	θ	1.7871	6.0095					
MKITL	α	1.4212	0.1359	0.1015	0.1272	0.7577	194.5589	199.1122
	β	1.1937	0.0725					
OWITL	α	1.8048	0.2146	0.0969	0.0873	0.5415	195.0995	201.9295
	β	25.9044	64.9941					
	λ	0.2721	0.3106					

**Table 7:** MLE with SE and other metrics: Group 1.

		Estimation	SE	KS	CVM	AD	AIC	BIC
ITL	δ	1.5750	0.4981	0.3141	0.0984	0.5089	41.4301	41.7327
NEITL	θ	0.4244	0.4225	**0.2129**	**0.0924**	**0.4806**	**41.4921**	**42.0973**
	δ	5.6463	5.1060					
EL	α	2.6559	2.0932	0.2181	0.0928	0.4831	43.5499	44.4577
	β	3.4714	4.0843					
	λ	3.4753	6.7246					
ExEx	α	1.0925	0.7304	0.2348	0.1133	0.6265	43.3593	43.9644
	β	0.3309	0.3558					
W	α	1.1585	0.2641	0.5557	0.0968	0.5082	42.9958	43.6010
	β	0.3042	0.1512					
KW	α	1.7884	2.5890	0.2187	0.0959	0.4999	45.6162	46.8265
	β	0.4544	1.7933					
	λ	8.9793	52.9562					
	θ	1.3099	10.1035					
KITL	α	1.5511	0.5350	0.2242	0.0944	0.4895	43.5807	44.4885
	β	10.1497	9.1324					
	λ	0.3556	1.1931					
MKITL	α	1.0353	0.2709	0.2523	0.1036	0.5577	42.1597	42.7649
	β	0.9040	0.1948					
OWITL	α	1.3963	0.3356	0.2173	0.0943	0.4919	43.5921	44.4998
	β	55.1455	193.5675					
	λ	0.0780	0.1781					

**Table 8:** MLE with SE and other metrics: Group 2.

		Estimates	SE	KS	CVM	AD	AIC	BIC
ITL	δ	2.0929	0.6618	0.2130	0.0520	0.2973	32.7512	33.0537
NEITL	θ	0.8260	1.5968	**0.2018**	**0.0405**	**0.2400**	**31.6464**	**32.2516**
	δ	3.8521	8.1014					
EL	α	7.0316	22.8576	0.2174	0.0527	0.2983	33.3300	34.2377
	β	3.1443	7.0486					
	λ	1.1988	6.2228					
ExEx	α	6.3206	23.1429	0.1789	0.0621	0.4414	33.7072	34.3124
	β	0.0589	0.2375					
W	α	1.5527	0.3675	0.7012	0.0505	0.3002	32.4143	33.0195
	β	0.3519	0.1683					
KW	α	7.7574	0.0025	0.2124	0.0411	0.2415	33.5910	34.8013
	β	0.9910	0.0025					
	λ	70.4798	59.0513					
	θ	0.1124	0.0366					
KITL	α	6.3918	26.4265	0.2233	0.0501	0.2878	33.0906	33.9984
	β	0.2378	0.8625					
	λ	12.4011	40.8768					

**Table 9:** MLE, MPS, and Bayeisan for reliability of the S–S model.

	MLE			MPS			Bayesian		
	**Estimates**	**SE**	**R**	**Estimates**	**SE**	**R**	**Estimates**	**SE**	**R**
$\theta_{1}$	0.4246	0.4226	0.6123	0.4143	0.4163	0.6345	0.5423	0.3812	0.6743
$\delta_{1}$	5.6440	5.1041		5.4284	4.9402		6.6363	4.5001	
$\theta_{2}$	0.8312	1.6372		0.8312	1.2626		1.1196	0.8512	
$\delta_{2}$	3.8267	8.2125		3.8267	7.4954		4.8956	3.8509	

## Data Availability

The data used to support the findings of this study are included within the article.
